# Post-COVID Condition in Patients With Cardiovascular Disease: Are Antivirals the Answer?∗

**DOI:** 10.1016/j.jacadv.2024.100962

**Published:** 2024-06-26

**Authors:** Maciej Dębski, Vasiliki Tsampasian, Vassilios S. Vassiliou

**Affiliations:** Norwich Medical School, University of East Anglia, Norwich, United Kingdom

**Keywords:** cardiovascular disease, healthcare utilization, long covid, nirmatrelvir-ritonavir, post-acute sequelae of SARS-CoV-2 infection

**Figure:**
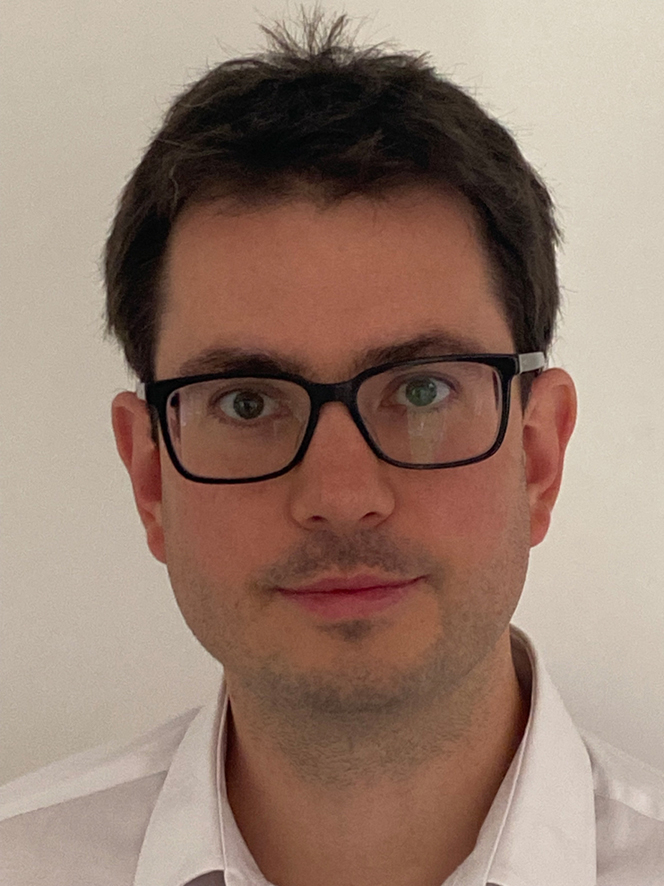

“Let food be thy medicine and medicine be thy food”

—Hippocrates

The COVID-19 pandemic has presented complex challenges to global health care systems.^1^, ^2^, ^3^ These challenges changed as new strains became dominant, vaccination arrived, and antivirals were prescribed for acute treatment in selected patients. The risk of mortality from acute infection has substantially decreased, especially in vaccinated individuals, but long-lasting symptoms related to COVID-19 infection continue to trouble individuals. Referred to as post-COVID conditions, long COVID, postacute sequelae of COVID-19 (PASC), or long-haul COVID, this syndrome has been defined by the World Health Organization as a condition seen in individuals with confirmed or presumed SARS-CoV-2 infection. Symptoms start within 3 months of an acute infection and last for at least 2 months without an alternative diagnosis to explain them. PASC affects around 5 to 10% of patients with COVID-19 and continues to affect millions of patients globally.^4^^,^^5^ PASC has attracted significant attention due to its debilitating and prolonged nature and high burden of health care service utilization.^6^^,^^7^ Vaccination prior to COVID-19 has been shown to reduce its incidence, while increasing age, obesity, female sex, and suffering from cardiac or respiratory conditions are shown to increase its incidence.^8^ Proposed contributing mechanisms, especially in patients with cardiovascular disease, include augmented immune response, persistent inflammation via T-cells, auto-antibodies, and pro-inflammatory cytokines, reactivation of latent viruses, and persistent SARS-CoV-2 infection.^9^ To date, there is no solid evidence that any pharmacotherapy administered during the acute infection phase could alter the incidence of PASC. However, a recent meta-analysis of observational studies supported that antiviral therapy could achieve a 25% reduction in PASC in all-comer patients, but the specific association in patients with known cardiac disease has not been investigated.^10^

It is against this background that we welcome the study by Patel et al^11^ on the role of nirmatrelvir-ritonavir in the acute phase of COVID-19 and subsequent incidence of PASC.

Nirmatrelvir-ritonavir is a COVID-19 oral antiviral treatment consisting of nirmatrelvir, a SARS-CoV-2 main protease inhibitor, and ritonavir, a pharmacokinetic enhancer. It was approved by the U.S. Food and Drug Administration for the treatment of mild-to-moderate COVID-19 in adults who are at high risk for progression to severe disease, including hospitalization or death.^12^ Patel et al undertook a retrospective cohort study in 26,594 nonhospitalized but vaccinated patients across 34 medical providers, treated with nirmatrelvir-ritonavir, and matched them to a cohort of patients who did not take nirmatrelvir-ritonavir. The authors should be congratulated for their study, which adds further evidence, albeit of an observational nature, for the potential benefit of nirmatrelvir-ritonavir regarding PASC in cardiac patients. They considered PASC at 2 time frames, 30 to 90 days and 90 to 180 days, using narrow and broad definitions of PASC. Independently of which comparison was made, there was an approximate 5% absolute reduction in the incidence of PASC, which was statistically significant and consistent across all clusters of symptoms. Furthermore, health care resource use such as radiology imaging, cardiovascular tests, and ambulatory/virtual visits was also reduced in the treated group by about 2% to 3%, resulting in additional cost benefits. It is also worth noting that the authors appeared to be free from any pharmaceutical conflict of interest with the company manufacturing nirmatrelvir-ritonavir.

With the conclusion of this study, should nirmatrelvir-ritonavir be prescribed for all patients with pre-existing cardiac conditions? Or, to paraphrase Hippocrates, is this just food for thought?

Firstly, as the authors acknowledge, given the observational design of this study, any causal relations cannot be presumed. The risk of residual confounding and selection bias cannot be overlooked. Secondly, the retrospective nature also meant that the diagnosis for PASC was based on self-reporting. Moreover, the heterogeneous nature of PASC poses challenges in precise characterization and, importantly, assessment of the severity of PASC. Not all patients are affected equally by PASC; some have a mildly affected quality of life while others have a significantly affected quality of life. This needs to be investigated to see whether the observed benefit from nirmatrelvir-ritonavir affects all PASC patients equally. Ideally, we would need to see nirmatrelvir-ritonavir reduce not only the incidence but also the severity and duration of PASC. In addition, health care providers need to be aware that nirmatrelvir-ritonavir has numerous drug-drug interactions, for instance, with statins, oral anticoagulants, certain antiarrhythmics, and antihypertensive medications, some of which require temporary hold or alteration of the dose.^13^ Finally, most of the patients with significant cardiac disease would already have been eligible for nirmatrelvir-ritonavir for the benefits seen in the acute infection. This study does not highlight an additional group that can receive it purely to reduce PASC severity.

Where do we go from this study? As new evidence emerges, eligible patients may be more keen to take nirmatrelvir-ritonavir. At present, those with pre-existing conditions who are eligible for the drug may choose not to take it. However, it would be appropriate to inform them that in observational studies, nirmatrelvir-ritonavir has been shown to reduce the incidence of PASC. Only then can patients make a fully informed decision.

On the research front, randomized studies looking at the use of nirmatrelvir-ritonavir in the acute phase of SARS-CoV-2 infection or in those suffering from long COVID already for the sole hypothesis of reducing the occurrence or severity of PASC are currently underway (PAxlovid loNg cOvid-19 pRevention triAl with recruitMent in the Community in Norway [PanoramicNOR], NCT05852873; RECOVER-VITAL: platform protocol to measure the effects of antiviral therapies on long COVID symptoms, NCT05595369; A decentralized, randomized phase 2 efficacy and safety study of nirmatrelvir/ritonavir in adults with long COVID, NCT05668091). Once those have been completed, then we can finally know the true value of nirmatrelvir-ritonavir in reducing PASC, but on the basis of the current observational data, it would seem that in the future, nirmatrelvir-ritonavir will have a role to play in PASC prevention.

## Funding support and author disclosures

Dr Dębski is supported by United Kingdom National Institute for Health and Care Research (NIHR) as a Clinical Lecturer, award number CL 2022-15-002. Dr Tsampasian is supported by an 10.13039/100006662NIHR Doctoral Research Fellowship award number 303306. Dr Vassiliou has reported that he has no relationships relevant to the contents of this paper to disclose.
